# Supplementary material for: Discordance between Invasive and Non-Invasive Coronary Angiography: An In-Depth Functional and Anatomical Analysis
Source: Biomedicines. 2023 Mar 15;11(3):913. doi: 10.3390/biomedicines11030913 (PMC10045446; doi:10.3390/biomedicines11030913)
Supplement: Supplementary file 1 [file biomedicines-11-00913-s001.zip › biomedicines-2199908-supplementary.pdf]

Supplemental Material

Methodology to calculate the lesion parameter in FFR_{CT}

Planar-area

This is the area of the cross-section of a lumen contour that is perpendicular to the centerline at that centerline point. Using this method, we could obtain reference area and minimum lumen area. See image below:

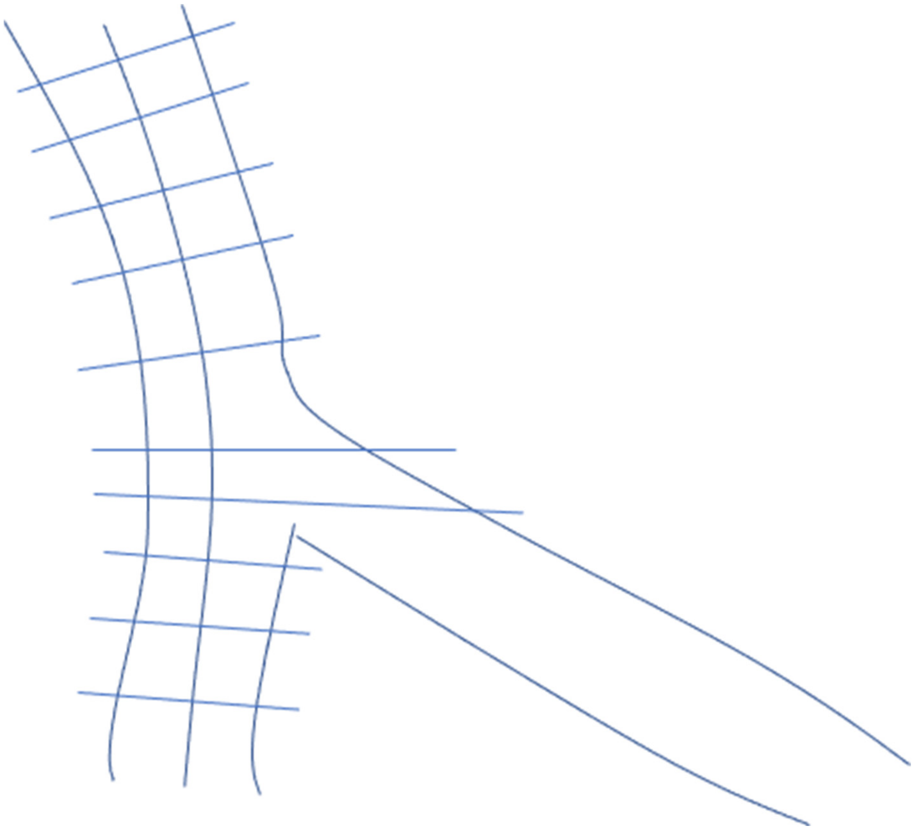

Sphere-inscribed-radius

This is the radius of the sphere that is completely enclosed or does not extend past any part of the lumen at that centerline point. Using this methodology, we could obtain reference diameter and minimum lumen diameter. See image below:

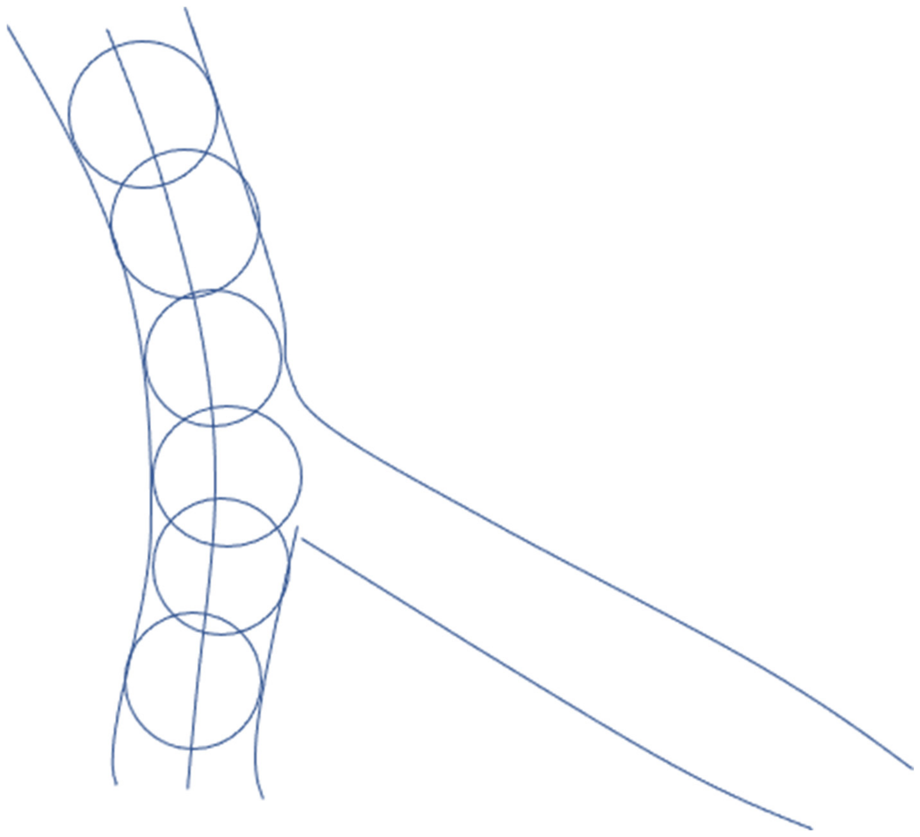

Lumen-narrowing-score

HeartFlow computes “idealized” lumen, which represents an estimate of the lumen without disease. Lumen narrowing score uses the sphere inscribed radius and this idealized radius to compute the value which has very similar concept to percent diameter stenosis. It is defined as 1 - (sphere inscribed radius/idealized radius). In the manuscript, we compared this value to percent diameter stenosis obtained from other modalities.
